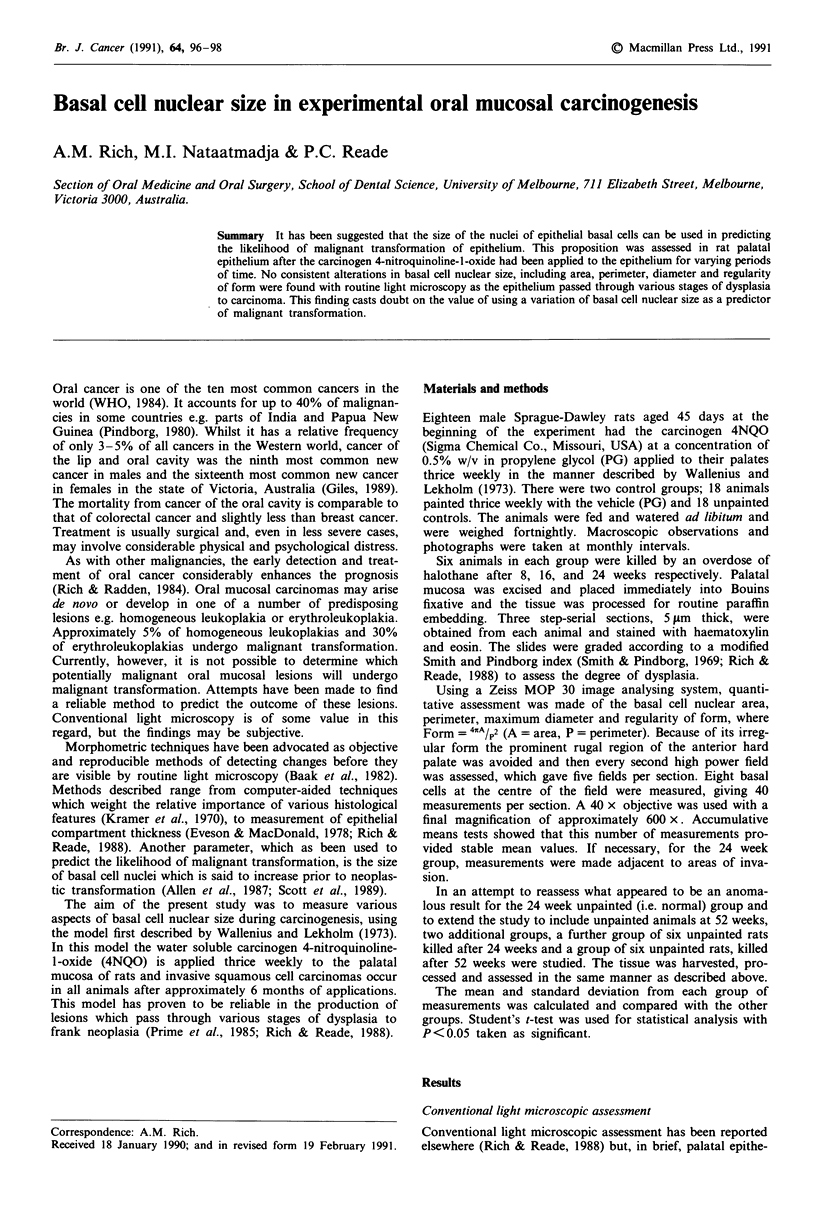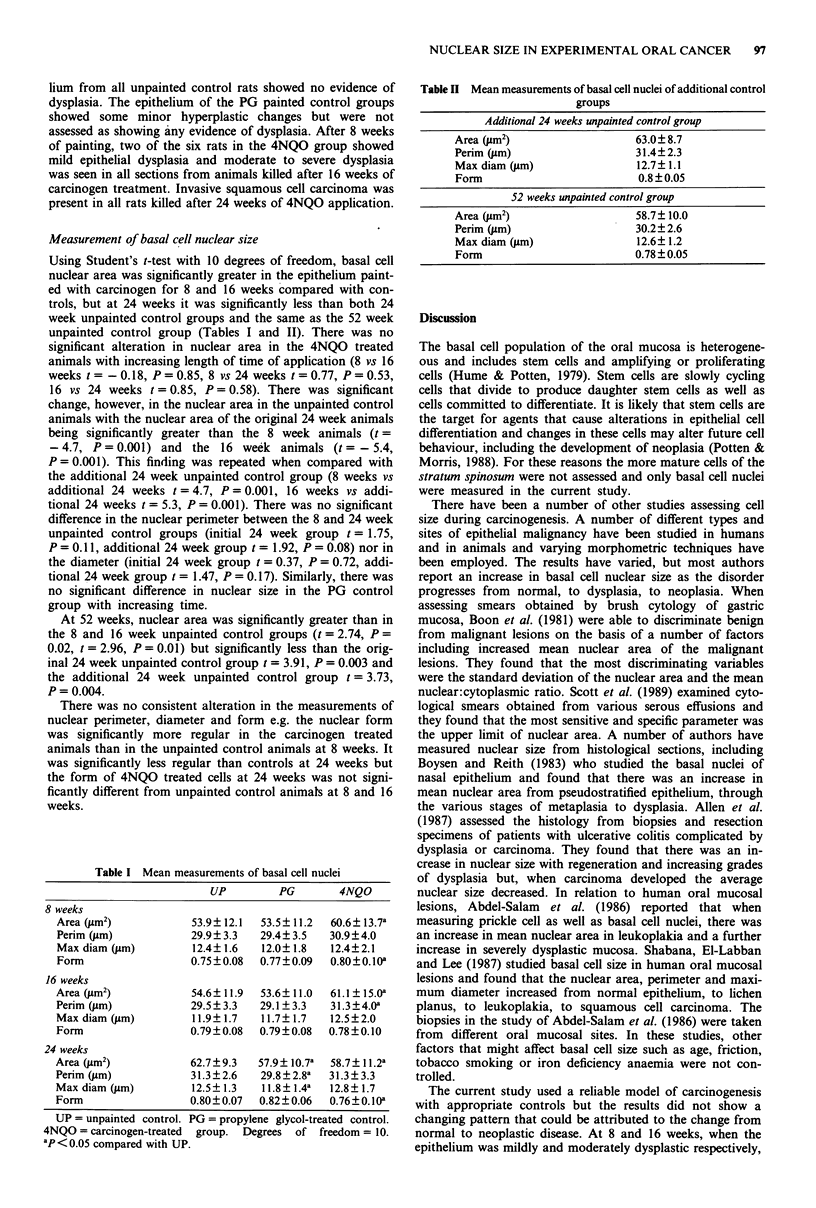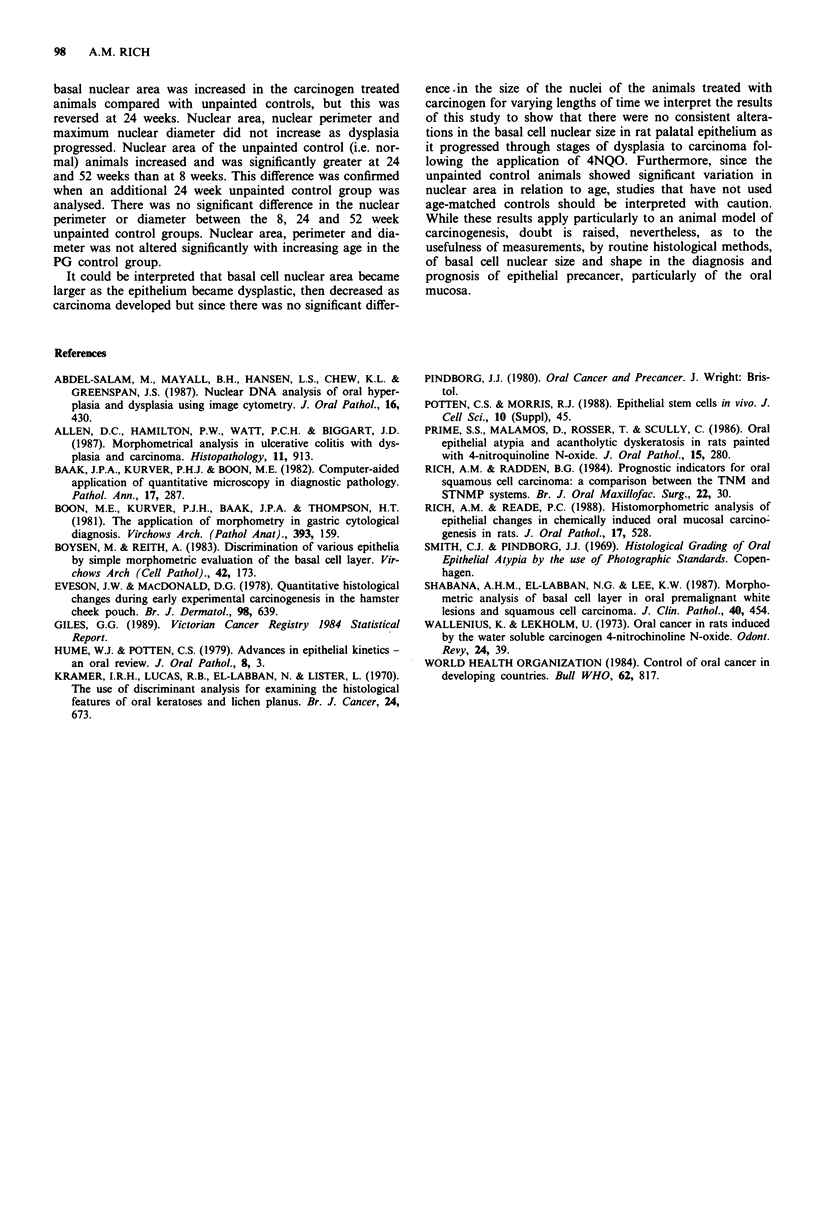# Basal cell nuclear size in experimental oral mucosal carcinogenesis.

**DOI:** 10.1038/bjc.1991.248

**Published:** 1991-07

**Authors:** A. M. Rich, M. I. Nataatmadja, P. C. Reade

**Affiliations:** Section of Oral Medicine and Oral Surgery, School of Dental Science, University of Melbourne, Victoria, Australia.

## Abstract

It has been suggested that the size of the nuclei of epithelial basal cells can be used in predicting the likelihood of malignant transformation of epithelium. This proposition was assessed in rat palatal epithelium after the carcinogen 4-nitroquinoline-1-oxide had been applied to the epithelium for varying periods of time. No consistent alterations in basal cell nuclear size, including area, perimeter, diameter and regularity of form were found with routine light microscopy as the epithelium passed through various stages of dysplasia to carcinoma. This finding casts doubt on the value of using a variation of basal cell nuclear size as a predictor of malignant transformation.


*Br. J. Cancer* (1991), **64**, 96–98 © Macmillan Press Ltd., 1991

### Basal cell nuclear size in experimental oral mucosal carcinogenesis

A.M. Rich, M.I. Nataatmadja & P.C. Reade

*Section of Oral Medicine and Oral Surgery, School of Dental Science, University of Melbourne, 711 Elizabeth Street, Melbourne, Victoria 3000, Australia.*

**Summary** It has been suggested that the size of the nuclei of epithelial basal cells can be used in predicting the likelihood of malignant transformation of epithelium. This proposition was assessed in rat palatal epithelium after the carcinogen 4-nitroquinoline-1-oxide had been applied to the epithelium for varying periods of time. No consistent alterations in basal cell nuclear size, including area, perimeter, diameter and regularity of form were found with routine light microscopy as the epithelium passed through various stages of dysplasia to carcinoma. This finding casts doubt on the value of using a variation of basal cell nuclear size as a predictor of malignant transformation.

Oral cancer is one of the ten most common cancers in the world (WHO, 1984). It accounts for up to 40% of malignancies in some countries e.g. parts of India and Papua New Guinea (Pindborg, 1980). Whilst it has a relative frequency of only 3–5% of all cancers in the Western world, cancer of the lip and oral cavity was the ninth most common new cancer in males and the sixteenth most common new cancer in females in the state of Victoria, Australia (Giles, 1989). The mortality from cancer of the oral cavity is comparable to that of colorectal cancer and slightly less than breast cancer. Treatment is usually surgical and, even in less severe cases, may involve considerable physical and psychological distress.

As with other malignancies, the early detection and treatment of oral cancer considerably enhances the prognosis (Rich & Radden, 1984). Oral mucosal carcinomas may arise *de novo* or develop in one of a number of predisposing lesions e.g. homogeneous leukoplakia or erythroleukoplakia. Approximately 5% of homogeneous leukoplakias and 30% of erythroleukoplakias undergo malignant transformation. Currently, however, it is not possible to determine which potentially malignant oral mucosal lesions will undergo malignant transformation. Attempts have been made to find a reliable method to predict the outcome of these lesions. Conventional light microscopy is of some value in this regard, but the findings may be subjective.

Morphometric techniques have been advocated as objective and reproducible methods of detecting changes before they are visible by routine light microscopy (Baak *et al.*, 1982). Methods described range from computer-aided techniques which weight the relative importance of various histological features (Kramer *et al.*, 1970), to measurement of epithelial compartment thickness (Eveson & MacDonald, 1978; Rich & Reade, 1988). Another parameter, which as been used to predict the likelihood of malignant transformation, is the size of basal cell nuclei which is said to increase prior to neoplastic transformation (Allen *et al.*, 1987; Scott *et al.*, 1989).

The aim of the present study was to measure various aspects of basal cell nuclear size during carcinogenesis, using the model first described by Wallenius and Lekholm (1973). In this model the water soluble carcinogen 4-nitroquinoline-1-oxide (4NQO) is applied thrice weekly to the palatal mucosa of rats and invasive squamous cell carcinomas occur in all animals after approximately 6 months of applications. This model has proven to be reliable in the production of lesions which pass through various stages of dysplasia to frank neoplasia (Prime *et al.*, 1985; Rich & Reade, 1988).

Correspondence: A.M. Rich.
Received 18 January 1990; and in revised form 19 February 1991.

### Materials and methods

Eighteen male Sprague-Dawley rats aged 45 days at the beginning of the experiment had the carcinogen 4NQO (Sigma Chemical Co., Missouri, USA) at a concentration of 0.5% w/v in propylene glycol (PG) applied to their palates thrice weekly in the manner described by Wallenius and Lekholm (1973). There were two control groups; 18 animals painted thrice weekly with the vehicle (PG) and 18 unpainted controls. The animals were fed and watered *ad libitum* and were weighed fortnightly. Macroscopic observations and photographs were taken at monthly intervals.

Six animals in each group were killed by an overdose of halothane after 8, 16, and 24 weeks respectively. Palatal mucosa was excised and placed immediately into Bouins fixative and the tissue was processed for routine paraffin embedding. Three step-serial sections, 5 $\mu$m thick, were obtained from each animal and stained with haematoxylin and eosin. The slides were graded according to a modified Smith and Pindborg index (Smith & Pindborg, 1969; Rich & Reade, 1988) to assess the degree of dysplasia.

Using a Zeiss MOP 30 image analysing system, quantitative assessment was made of the basal cell nuclear area, perimeter, maximum diameter and regularity of form, where Form = $^{4\pi A}/_{P^2}$ (A = area, P = perimeter). Because of its irregular form the prominent rugal region of the anterior hard palate was avoided and then every second high power field was assessed, which gave five fields per section. Eight basal cells at the centre of the field were measured, giving 40 measurements per section. A 40 × objective was used with a final magnification of approximately 600 ×. Accumulative means tests showed that this number of measurements provided stable mean values. If necessary, for the 24 week group, measurements were made adjacent to areas of invasion.

In an attempt to reassess what appeared to be an anomalous result for the 24 week unpainted (i.e. normal) group and to extend the study to include unpainted animals at 52 weeks, two additional groups, a further group of six unpainted rats killed after 24 weeks and a group of six unpainted rats, killed after 52 weeks were studied. The tissue was harvested, processed and assessed in the same manner as described above.

The mean and standard deviation from each group of measurements was calculated and compared with the other groups. Student's *t*-test was used for statistical analysis with $P < 0.05$ taken as significant.

### Results

### *Conventional light microscopic assessment*

Conventional light microscopic assessment has been reported elsewhere (Rich & Reade, 1988) but, in brief, palatal epithelium from all unpainted control rats showed no evidence of dysplasia. The epithelium of the PG painted control groups showed some minor hyperplastic changes but were not assessed as showing any evidence of dysplasia. After 8 weeks of painting, two of the six rats in the 4NQO group showed mild epithelial dysplasia and moderate to severe dysplasia was seen in all sections from animals killed after 16 weeks of carcinogen treatment. Invasive squamous cell carcinoma was present in all rats killed after 24 weeks of 4NQO application.

### *Measurement of basal cell nuclear size*

Using Student's $t$-test with 10 degrees of freedom, basal cell nuclear area was significantly greater in the epithelium painted with carcinogen for 8 and 16 weeks compared with controls, but at 24 weeks it was significantly less than both 24 week unpainted control groups and the same as the 52 week unpainted control group (Tables I and II). There was no significant alteration in nuclear area in the 4NQO treated animals with increasing length of time of application (8 *vs* 16 weeks $t = -0.18$, $P = 0.85$, 8 *vs* 24 weeks $t = 0.77$, $P = 0.53$, 16 *vs* 24 weeks $t = 0.85$, $P = 0.58$). There was significant change, however, in the nuclear area in the unpainted control animals with the nuclear area of the original 24 week animals being significantly greater than the 8 week animals ($t = -4.7$, $P = 0.001$) and the 16 week animals ($t = -5.4$, $P = 0.001$). This finding was repeated when compared with the additional 24 week unpainted control group (8 weeks *vs* additional 24 weeks $t = 4.7$, $P = 0.001$, 16 weeks *vs* additional 24 weeks $t = 5.3$, $P = 0.001$). There was no significant difference in the nuclear perimeter between the 8 and 24 week unpainted control groups (initial 24 week group $t = 1.75$, $P = 0.11$, additional 24 week group $t = 1.92$, $P = 0.08$) nor in the diameter (initial 24 week group $t = 0.37$, $P = 0.72$, additional 24 week group $t = 1.47$, $P = 0.17$). Similarly, there was no significant difference in nuclear size in the PG control group with increasing time.

At 52 weeks, nuclear area was significantly greater than in the 8 and 16 week unpainted control groups ($t = 2.74$, $P = 0.02$, $t = 2.96$, $P = 0.01$) but significantly less than the original 24 week unpainted control group $t = 3.91$, $P = 0.003$ and the additional 24 week unpainted control group $t = 3.73$, $P = 0.004$.

There was no consistent alteration in the measurements of nuclear perimeter, diameter and form e.g. the nuclear form was significantly more regular in the carcinogen treated animals than in the unpainted control animals at 8 weeks. It was significantly less regular than controls at 24 weeks but the form of 4NQO treated cells at 24 weeks was not significantly different from unpainted control animals at 8 and 16 weeks.

**Table I** Mean measurements of basal cell nuclei

| | *UP* | *PG* | *4NQO* |
|---|---|---|---|
| *8 weeks* | | | |
| Area (μm²) | 53.9±12.1 | 53.5±11.2 | 60.6±13.7[a] |
| Perim (μm) | 29.9±3.3 | 29.4±3.5 | 30.9±4.0 |
| Max diam (μm) | 12.4±1.6 | 12.0±1.8 | 12.4±2.1 |
| Form | 0.75±0.08 | 0.77±0.09 | 0.80±0.10[a] |
| *16 weeks* | | | |
| Area (μm²) | 54.6±11.9 | 53.6±11.0 | 61.1±15.0[a] |
| Perim (μm) | 29.5±3.3 | 29.1±3.3 | 31.3±4.0[a] |
| Max diam (μm) | 11.9±1.7 | 11.7±1.7 | 12.5±2.0 |
| Form | 0.79±0.08 | 0.79±0.08 | 0.78±0.10 |
| *24 weeks* | | | |
| Area (μm²) | 62.7±9.3 | 57.9±10.7[a] | 58.7±11.2[a] |
| Perim (μm) | 31.3±2.6 | 29.8±2.8[a] | 31.3±3.3 |
| Max diam (μm) | 12.5±1.3 | 11.8±1.4[a] | 12.8±1.7 |
| Form | 0.80±0.07 | 0.82±0.06 | 0.76±0.10[a] |

UP = unpainted control. PG = propylene glycol-treated control. 4NQO = carcinogen-treated group. Degrees of freedom = 10. [a]$P < 0.05$ compared with UP.

**Table II** Mean measurements of basal cell nuclei of additional control groups

| | |
|---|---|
| *Additional 24 weeks unpainted control group* | |
| Area (μm²) | 63.0±8.7 |
| Perim (μm) | 31.4±2.3 |
| Max diam (μm) | 12.7±1.1 |
| Form | 0.8±0.05 |
| *52 weeks unpainted control group* | |
| Area (μm²) | 58.7±10.0 |
| Perim (μm) | 30.2±2.6 |
| Max diam (μm) | 12.6±1.2 |
| Form | 0.78±0.05 |

### Discussion

The basal cell population of the oral mucosa is heterogeneous and includes stem cells and amplifying or proliferating cells (Hume & Potten, 1979). Stem cells are slowly cycling cells that divide to produce daughter stem cells as well as cells committed to differentiate. It is likely that stem cells are the target for agents that cause alterations in epithelial cell differentiation and changes in these cells may alter future cell behaviour, including the development of neoplasia (Potten & Morris, 1988). For these reasons the more mature cells of the *stratum spinosum* were not assessed and only basal cell nuclei were measured in the current study.

There have been a number of other studies assessing cell size during carcinogenesis. A number of different types and sites of epithelial malignancy have been studied in humans and in animals and varying morphometric techniques have been employed. The results have varied, but most authors report an increase in basal cell nuclear size as the disorder progresses from normal, to dysplasia, to neoplasia. When assessing smears obtained by brush cytology of gastric mucosa, Boon *et al.* (1981) were able to discriminate benign from malignant lesions on the basis of a number of factors including increased mean nuclear area of the malignant lesions. They found that the most discriminating variables were the standard deviation of the nuclear area and the mean nuclear:cytoplasmic ratio. Scott *et al.* (1989) examined cytological smears obtained from various serous effusions and they found that the most sensitive and specific parameter was the upper limit of nuclear area. A number of authors have measured nuclear size from histological sections, including Boysen and Reith (1983) who studied the basal nuclei of nasal epithelium and found that there was an increase in mean nuclear area from pseudostratified epithelium, through the various stages of metaplasia to dysplasia. Allen *et al.* (1987) assessed the histology from biopsies and resection specimens of patients with ulcerative colitis complicated by dysplasia or carcinoma. They found that there was an increase in nuclear size with regeneration and increasing grades of dysplasia but, when carcinoma developed the average nuclear size decreased. In relation to human oral mucosal lesions, Abdel-Salam *et al.* (1986) reported that when measuring prickle cell as well as basal cell nuclei, there was an increase in mean nuclear area in leukoplakia and a further increase in severely dysplastic mucosa. Shabana, El-Labban and Lee (1987) studied basal cell size in human oral mucosal lesions and found that the nuclear area, perimeter and maximum diameter increased from normal epithelium, to lichen planus, to leukoplakia, to squamous cell carcinoma. The biopsies in the study of Abdel-Salam *et al.* (1986) were taken from different oral mucosal sites. In these studies, other factors that might affect basal cell size such as age, friction, tobacco smoking or iron deficiency anaemia were not controlled.

The current study used a reliable model of carcinogenesis with appropriate controls but the results did not show a changing pattern that could be attributed to the change from normal to neoplastic disease. At 8 and 16 weeks, when the epithelium was mildly and moderately dysplastic respectively, basal nuclear area was increased in the carcinogen treated animals compared with unpainted controls, but this was reversed at 24 weeks. Nuclear area, nuclear perimeter and maximum nuclear diameter did not increase as dysplasia progressed. Nuclear area of the unpainted control (i.e. normal) animals increased and was significantly greater at 24 and 52 weeks than at 8 weeks. This difference was confirmed when an additional 24 week unpainted control group was analysed. There was no significant difference in the nuclear perimeter or diameter between the 8, 24 and 52 week unpainted control groups. Nuclear area, perimeter and diameter was not altered significantly with increasing age in the PG control group.

It could be interpreted that basal cell nuclear area became larger as the epithelium became dysplastic, then decreased as carcinoma developed but since there was no significant difference in the size of the nuclei of the animals treated with carcinogen for varying lengths of time we interpret the results of this study to show that there were no consistent alterations in the basal cell nuclear size in rat palatal epithelium as it progressed through stages of dysplasia to carcinoma following the application of 4NQO. Furthermore, since the unpainted control animals showed significant variation in nuclear area in relation to age, studies that have not used age-matched controls should be interpreted with caution. While these results apply particularly to an animal model of carcinogenesis, doubt is raised, nevertheless, as to the usefulness of measurements, by routine histological methods, of basal cell nuclear size and shape in the diagnosis and prognosis of epithelial precancer, particularly of the oral mucosa.

### References

ABDEL-SALAM, M., MAYALL, B.H., HANSEN, L.S., CHEW, K.L. & GREENSPAN, J.S. (1987). Nuclear DNA analysis of oral hyperplasia and dysplasia using image cytometry. *J. Oral Pathol.*, **16**, 430.

ALLEN, D.C., HAMILTON, P.W., WATT, P.C.H. & BIGGART, J.D. (1987). Morphometrical analysis in ulcerative colitis with dysplasia and carcinoma. *Histopathology*, **11**, 913.

BAAK, J.P.A., KURVER, P.H.J. & BOON, M.E. (1982). Computer-aided application of quantitative microscopy in diagnostic pathology. *Pathol. Ann.*, **17**, 287.

BOON, M.E., KURVER, P.J.H., BAAK, J.P.A. & THOMPSON, H.T. (1981). The application of morphometry in gastric cytological diagnosis. *Virchows Arch. (Pathol Anat).*, **393**, 159.

BOYSEN, M. & REITH, A. (1983). Discrimination of various epithelia by simple morphometric evaluation of the basal cell layer. *Virchows Arch (Cell Pathol).*, **42**, 173.

EVESON, J.W. & MACDONALD, D.G. (1978). Quantitative histological changes during early experimental carcinogenesis in the hamster cheek pouch. *Br. J. Dermatol.*, **98**, 639.

GILES, G.G. (1989). *Victorian Cancer Registry 1984 Statistical Report*.

HUME, W.J. & POTTEN, C.S. (1979). Advances in epithelial kinetics – an oral review. *J. Oral Pathol.*, **8**, 3.

KRAMER, I.R.H., LUCAS, R.B., EL-LABBAN, N. & LISTER, L. (1970). The use of discriminant analysis for examining the histological features of oral keratoses and lichen planus. *Br. J. Cancer*, **24**, 673.

PINDBORG, J.J. (1980). *Oral Cancer and Precancer*. J. Wright: Bristol.

POTTEN, C.S. & MORRIS, R.J. (1988). Epithelial stem cells *in vivo*. *J. Cell Sci.*, **10** (Suppl), 45.

PRIME, S.S., MALAMOS, D., ROSSER, T. & SCULLY, C. (1986). Oral epithelial atypia and acantholytic dyskeratosis in rats painted with 4-nitroquinoline N-oxide. *J. Oral Pathol.*, **15**, 280.

RICH, A.M. & RADDEN, B.G. (1984). Prognostic indicators for oral squamous cell carcinoma: a comparison between the TNM and STNMP systems. *Br. J. Oral Maxillofac. Surg.*, **22**, 30.

RICH, A.M. & READE, P.C. (1988). Histomorphometric analysis of epithelial changes in chemically induced oral mucosal carcinogenesis in rats. *J. Oral Pathol.*, **17**, 528.

SMITH, C.J. & PINDBORG, J.J. (1969). *Histological Grading of Oral Epithelial Atypia by the use of Photographic Standards*. Copenhagen.

SHABANA, A.H.M., EL-LABBAN, N.G. & LEE, K.W. (1987). Morphometric analysis of basal cell layer in oral premalignant white lesions and squamous cell carcinoma. *J. Clin. Pathol.*, **40**, 454.

WALLENIUS, K. & LEKHOLM, U. (1973). Oral cancer in rats induced by the water soluble carcinogen 4-nitrochinoline N-oxide. *Odont. Revy*, **24**, 39.

WORLD HEALTH ORGANIZATION (1984). Control of oral cancer in developing countries. *Bull WHO*, **62**, 817.